# Legitimacy as Social Infrastructure: A Critical Interpretive Synthesis of the Literature on Legitimacy in Health and Technology

**DOI:** 10.2196/48955

**Published:** 2025-03-05

**Authors:** Sydney Howe, Carin Uyl-de Groot, Rik Wehrens

**Affiliations:** 1 Erasmus School of Health Policy and Management Erasmus University Rotterdam Rotterdam The Netherlands

**Keywords:** legitimacy, health technology, infrastructure, literature review, technology adoption, health care governance, technology acceptance, health care delivery, social infrastructure, critical interpretive synthesis

## Abstract

**Background:**

As technology is integrated into health care delivery, research on adoption and acceptance of health technologies leaves large gaps in practice and provides limited explanation of how and why certain technologies are adopted and others are not. In these discussions, the concept of legitimacy is omnipresent but often implicit and underdeveloped. There is no agreement about what legitimacy is or how it works across social science disciplines, despite a prolific volume of the literature centering legitimacy.

**Objective:**

This study aims to explore the meaning of legitimacy in health and technology as conceptualized in the distinctive disciplines of organization and management studies, science and technology studies, and medical anthropology and sociology, including how legitimacy is produced and used. This allows us to critically combine insights across disciplines and generate new theory.

**Methods:**

We conducted a critical interpretive synthesis literature review. Searches were conducted iteratively and were guided by preset eligibility criteria determined through thematic analysis, beginning with the selection of disciplines, followed by journals, and finally articles. We selected disciplines and journals in organization and management studies, science and technology studies, and medical anthropology and sociology using results from the Scopus and Web of Science databases and disciplinary expert–curated journal lists, focusing on the depth of legitimacy conceptualization. We selected 30 journals, yielding 796 abstracts.

**Results:**

A total of 97 articles were included. The synthesis of the literature allowed us to produce a novel conceptualization of legitimacy as a form of social infrastructure, approaching legitimacy as a binding fabric of relationships, narratives, and materialities. We argue that the notion of legitimacy as social infrastructure is a flexible and adaptable framework for working with legitimacy both theoretically and practically.

**Conclusions:**

The legitimacy as social infrastructure framework can aid both academics and decision makers by providing more coherent and holistic explanations for how and why new technologies are adopted or not in health care practice. For academics, our framework makes legitimacy and technology adoption empirically approachable from an ethnographic perspective; for decision makers, legitimacy as social infrastructure allows for a practical, action-oriented focus that can be assessed iteratively at any stage of the technology development and implementation process.

## Introduction

### Background

Technological innovations and health care delivery have become increasingly intertwined in recent decades [1,2]. With each promising innovation, new questions are raised about how to integrate the technology into health domains and the social implications of doing so [2-8]. This problem has given rise to evaluation processes such as health technology assessment [9] and a range of government institutions, such as the National Institute for Health and Care Excellence in the United Kingdom, to help make decisions about the appropriate use of technologies in various health care contexts [10].

Questions of legitimacy are implicit in debates about embedding technologies in health care, and these implicit questions about legitimacy permeate the literature about health and technology. While there is limited conceptual work on legitimacy in health and technology specifically, within the small body of work that exists, legitimacy is explored through a wide range of theoretical lenses and conceptualizations. However, there is no consensus about what legitimacy is or how it works nor is there much conversation among different branches of the literature. Across social science disciplines and medicine, legitimacy is affiliated with many concepts, including “acceptability” [11], “reasonableness” [12], and “transparency” [3,4]; these terms are sometimes conflated with legitimacy itself. Definitions of legitimacy range just as widely, especially among different disciplines: in organization and management studies (OMS), legitimacy is often defined as “normative appropriateness” [13,14], although science and technology studies (STS) and medical anthropology and sociology (MAS) include definitions such as “the acceptability of claims to authority” [15,16] and “accountability for reasonableness” [10] when the term is defined. Some disciplines, such as OMS, follow a strong tradition of explicit theorization of legitimacy, while others, such as medicine, usually do not explicitly problematize or define the word “legitimacy,” although legitimacy and similar concepts may play a substantial role in discussions in the field. Within the same discipline, some authors use legitimacy interchangeably with other concepts [6], while others focus on a single subcategory of legitimacy developed from a narrow line of theoretical thought [17]. Given the breadth and depth of the literature on legitimacy in health and technology, it is clearly an important topic. A cross-disciplinary conceptualization of legitimacy that can be applied to questions about embedding technologies in health care would allow for a deeper understanding of these processes.

We chose to focus on legitimacy for several reasons. Legitimacy specifically is widely used but poorly defined within multiple fields studying health and technology and points toward social and organizational processes. Even though legitimacy is clearly important in studies of health and technology across many disciplines, it is abstract and there is very little cohesion both within and across social science disciplines around how it is produced and how it is used.

To understand the role of legitimacy in relation to the embedding and governance of technology in health care, we fleshed out the different ideas and underlying assumptions authors bring to the OMS, STS, and MAS literature. This paper explores three research questions: (1) What does legitimacy mean in the context of health and technology? (2) How is legitimacy produced? (3) How is legitimacy used to explain the challenges of embedding technologies in health care? On the basis of a synthesis of the literature, we developed a novel conceptualization of legitimacy as social infrastructure. This allowed us to focus on the aspects of legitimacy related to norms, materialities, semiotics, and specific relationships among stakeholders and larger systemic contexts*.*

Existing frameworks guiding policy intentions around technology adoption in health care often do not reflect the realities of everyday practice nor do they focus on the social processes of embedding technologies in health care [18]. Current models and frameworks for embedding technologies (in health care), such as the Unified Theory of Acceptance and Use of Technology [19] and the Technology Acceptance Model (TAM) [20,21], provide useful information about the judgments and perceptions of various actors in relation to a specific technology. While these models provide useful insights into actors’ *intention* to adopt, as well as the ethical considerations they may perceive and institutional norms they may face, they do not often explain how and why certain technologies are or are not adopted or accepted *in practice* among particular groups and thus can provide only limited governance support [22]. Most technology adoption and acceptance models deal primarily with behavioral intention [20,21]. These models attempt to explain individual behavior intentions (as a proxy for actual behavior) through an analysis of a variety of factors, which can include beliefs, attitudes, perceived control, and perceived norms. This leaves substantial gaps, including the lack of attention to external factors impacting the embedding process [20] and any difference between behavioral intention and practice. Focusing on the impact of legitimacy within technology embedding processes can help fill these gaps by allowing for more comprehensive and nuanced conversations about technology adoption and acceptance.

### A Cross-Disciplinary Legitimacy Conceptualization

The goal of this review is to produce a theoretical synthesis of how the legitimacy of health and technology is conceptualized. We aim to generate theoretical insights that will allow for a cross-disciplinary approach to legitimacy. To do this, we used the critical interpretive synthesis (CIS) review method to produce a cross-disciplinary understanding [23] of how legitimacy is produced and maintained without privileging one discipline’s theoretical traditions over another’s [24].

We used OMS, STS, and MAS as a disciplinary framework to understand the different strands of reasoning within legitimacy studies. OMS includes the most clearly developed theorization of legitimacy as a concept and several of the best-known review articles [14,25]. Legitimacy studies is a well-defined subarea of OMS research that explores legitimacy in the context of management, entrepreneurship, and institutional endeavors. OMS primarily focuses on how legitimacy works at the level of organizations and institutions, rather than among individual people or through technologies.

STS explores the social and cultural implications of science, technology, and technology development [26,27]. STS tends to focus on controversies, the unforeseen implications of technology development and adoption, and “black box” technologies [28] that inhibit full understanding. This contributes to a strong disciplinary interest in legitimacy, particularly of technology and medicine. STS centers interactions with technologies and technical change in legitimacy discussions, rather than separating the motivations of human actors from technologies and practices.

Although MAS developed from different traditions, they have partially converged in the study of medical cultures and interactions from a qualitative perspective [29], which is also the focus of legitimacy studies in MAS. MAS generally takes a skeptical view of taken-for-granted institutions. Many studies explore the impact of this taken-for-grantedness on people who are harmed by or left out of these institutions and systems. This leads to a natural questioning of legitimacy and deeper exploration of baseline assumptions, particularly in a health care context. Together, these disciplines explore health and health technologies with less focus on institutions and more focus on humans and connections among them. We hope that greater synthesis of different conceptualizations of legitimacy could provide a more holistic perspective about how and why technologies are or are not embedded in health care that integrates ideas from outside current frameworks based on adoption, acceptance, and implementation.

This review does not focus on ethics, although some articles that deal with ethics are included in this review. Within ethics, there is general consensus that legitimacy in health and technology is produced through ethical judgments and values-based reasoning [30-33]. While there is more cohesion in ethics than in other disciplines that deal with legitimacy, the scope of the literature is narrower and generally limited to moral or ethical legitimacy [3,34]. Legitimacy is often defined in ethical terms across several disciplines, including management, STS, and health policy. Articles with an ethical focus on health care technology adoption usually prioritize normative and moral dimensions of legitimacy, which may or may not be related to other aspects that contribute to overall legitimacy, such as utility or social acceptability [35]. While moral legitimacy is an important dimension of the subject, it has also already been studied and reviewed extensively and is far more clearly defined within the literature than other dimensions of legitimacy. Therefore, we chose to focus on other aspects of legitimacy to fill gaps in the existing social science literature. In addition, our review does not center legal approaches. Legal literature defines legitimacy according to principles of a participatory democracy [36]. Because the focus of this review is on social processes rather than democratic principles, we have chosen to center other means of legitimacy production and thus other branches of the literature, while acknowledging that democratic principles can play an important role [15,37].

## Methods

### Overview

In a research landscape that increasingly encourages and necessitates interdisciplinary collaboration, a CIS [24] allows us to develop a richer conceptual frame that can bring these different disciplines into conversation with one another [23,38]. The CIS method specifically addresses the limitations of conventional systematic reviews in cases where the objective is to generate a critical analysis of a body of the literature [24]. It draws on a tradition of qualitative inquiry: rather than summarizing the literature using preidentified key concepts, CIS uses an inductive, iterative process to interpret data, develop key concepts, and synthesize concepts with data through the process of the review itself [39]. Therefore, CIS produces a critical interpretation of the data reported in literature, which allows questioning of taken-for-granted assumptions [24].

We found CIS to be a particularly appropriate review style for an exploration of legitimacy in health and technology because, unlike traditional review styles, CIS is adept at dealing with “a large, amorphous, and complex body of literature” [24]. CIS synthesizes findings in a way that is both conceptually valuable to researchers and useful in informing policy. Furthermore, the CIS review style can manage the abstract nature of legitimacy across 3 disciplines without reducing the complexity of existing literature.

CIS is methodological and rigorously structured. However, it is also labor intensive compared to other review styles and deprioritizes transparency and full replicability available through systematic reviews. As in other kinds of interpretive research, different researchers using the same materials may have come to different conclusions, and full transparency of analysis cannot be expected at the same level as quantitative studies. We chose the CIS method in service of greater interpretive flexibility and conceptual depth and because it allowed us to account for the diversity of qualitative research without privileging a small range of studies that met specific and, here, less-relevant methodological standards.

Despite many empirical papers that address some aspects of legitimacy in relation to health and technology, there is a much more limited number of articles dealing with legitimacy in the domains of health and technology at a conceptual level. Therefore, we adopted broad definitions of both technology and health, including as many articles with strong legitimacy theorization as possible. “Health” in this paper refers to “human health and well-being”; “technology” includes “any devices, machinery, or equipment developed for the application of scientific knowledge that may impact human health.” We acknowledge that there are potentially many other ways to operationalize these concepts that could be relevant to this review and health care in general [40]. Given the broad, conceptual focus of the review, as well as the wide range of distinctive technologies considered, we introduced 2 measures that allowed us to provide a clear focus. First, we zoomed in on conceptualizations of legitimacy; it was outside the scope to examine the specific empirical contexts and details of these conceptualizations (ie, the use of specific technologies within specific health practices). Second, looking at specific disciplines proved helpful to demarcate boundaries around our topic. We used academic disciplines as the basis of our theoretical categories to give structure to a broad and abstract review, following in a strong disciplinary synthesis tradition established by other CIS researchers [23,38].

Our review was conducted in 4 cycles [38]. In the first cycle, we conducted an exploratory search in relevant journals with many articles on legitimacy in the context of health and technology. Using this search and subsequent iterations, we refined inclusion criteria (for instance, a threshold for legitimacy theorization) to determine a list of disciplines, journals within each discipline, and articles within these journals that would be a part of our review (see Multimedia Appendix 1 for details). In the second cycle, we read and coded included articles directly from the text, then grouped codes into thematic areas. Third, we analyzed legitimacy in health and technology within each discipline through interpretive disciplinary summaries. Finally, we developed a synthesizing argument across all 3 disciplines, based on these interpretive summaries.

### Search Strategy

Journals within each discipline were selected for the review manually. Given the interdisciplinary nature of this review, we sought to include relevant journals within each discipline from a wide range to ensure diversity of perspectives.

The first author ran an initial search in Google Scholar and the Web of Science (WoS) database using the terms “legitimacy,” “health,” and “technology.” However, it became clear that regardless of how specific the search criteria became, most records returned through a database search would not include deep conceptualizations of the term “legitimacy.” This is because this term is commonly used across many disciplines as an unproblematic descriptor of technologies, legal processes, narrative choices, medical decisions, policies, and institutions. Therefore, we switched to a more iterative, manual strategy for discipline and subsequently journal selection (Textbox 1). The first author searched through a few of the major journals (based on impact factor or prior knowledge of the review team) in depth to understand whether studies addressing questions of legitimacy in a specific discipline generally included deeper conceptualization. This method allowed us to narrow down the disciplines to be included (see Multimedia Appendix 1 for details).

**Selected journals.**

**Organization and management studies**

- Journal of Business Venturing
- Journal of Management Studies
- Organization Studies
- Organization Science
- Administrative Science Quarterly
- Health Care Management Review
- Strategic Entrepreneurship Journal
- Health Care Analysis
- Strategic Organization
- European Journal of Public Health

**Science and technology studies**

- Science as Culture
- Science, Technology and Human Values
- Social Studies of Science
- Social Epistemology
- Journal of Responsible Innovation
- AI & Society
- Big Data and Society
- Risk Analysis
- Information Communication & Society
- Research Policy

**Medical anthropology and sociology**

- Medical Anthropology
- Medical Anthropology Quarterly
- Anthropology & Medicine
- Qualitative Health Research
- Critical Public Health
  (interdisciplinary journal: some articles included within science and technology studies)
- Sociology of Health & Illness
- Health: An Interdisciplinary Journal for the Social Study of Health, Illness and Medicine
- Social Science and Medicine
  (interdisciplinary journal: some articles included within science and technology studies)
- Sociology-The Journal of the British Sociological Association
- Sociological Theory

Upon the selection of disciplines, journals were first included based on impact factor: the top 10 journals in each discipline according to the WoS formed the base of the list. Then these journals were screened for topical relevance, and lower-ranked journals were excluded when the topic was not relevant. Finally, journals were added from library guides created by universities that are well-known in the field, especially if no definitive rankings were available from the WoS (STS) or there was too much disciplinary overlap in the rankings (MAS). This resulted in a list of approximately 20 journals per discipline. We then screened all journals for legitimacy, health, and technology results to produce the final list, prioritizing journals that contained significant theorization and conceptual work around legitimacy (see Multimedia Appendix 1 for details).

### Selection Process

#### Overview

**Figure 1:**
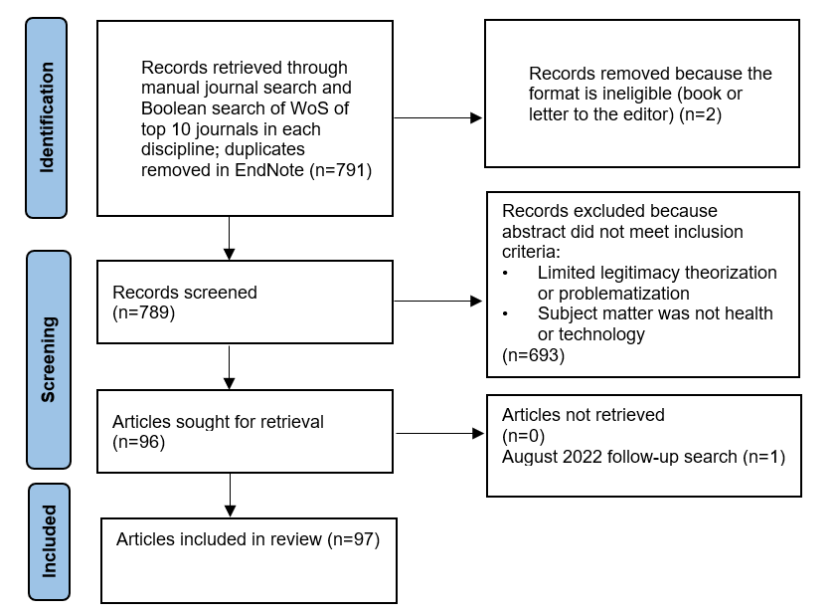
PRISMA (Preferred Reporting Items for Systematic Reviews and Meta-Analyses) flow diagram of inclusions. WoS: Web of Science.

A total of 791 abstracts were selected for initial review in April 2021 (Figure 1). Articles were sorted based on inclusion criteria: an article was included if there was evidence of explicit theorization or problematization of legitimacy in the abstract and the subject of the article was health or technology or both. A final search for more recent articles was run in August 2022, resulting in 1 additional inclusion. Multimedia Appendix 1 gives more information about the inclusion criteria.

#### Triangulation

The first author (SH) read all 791 abstracts and categorized them according to the inclusion criteria as “include,” “exclude,” or “review.” The last author (RW reviewed 79/791, 10% of the total articles’ abstracts at random (every 10th article when arranged in alphabetical order by author name). The resulting inclusion list closely matched SH’s selection. In the 7 disagreements, the 2 reviewers came to a joint decision about each contested article and edited the inclusion criteria. SH then reviewed the abstracts again. With no disagreements following this protocol, the literature list of 96 articles was finalized in March 2021, with an addition of 1 article in August 2022, bringing the total to 97, including, by discipline, 48 for STS, 29 for OMS, and 20 for MAS [4-8,10,12,13,15,16,37,41-126].

### Synthesis Methods

The goal of our initial analysis was to develop “synthetizing constructs” for each discipline, which “build on the explanations and interpretations of the constituent studies and are simultaneously consistent with the original results while extending beyond them” [24]. To accomplish this, SH first coded themes across a random selection of 30 articles spread equally across the 3 disciplines in Atlas.ti (ATLAS.ti Scientific Software Development GmbH). Inductive coding began with words taken from the article text. We made an exception for deductive codes relating to assumptions made as part of our “critical and reflexive approach” to this review [24]; for instance, coding whether articles discussed legitimacy as a process, perception, or asset based on our background understanding of existing legitimacy theorization across multiple disciplines. SH grouped codes by theme within each discipline. This thematic analysis was performed with the help of tables of code co-occurrences, generated in Atlas.ti, which showed overlap of unique codes within each document; this allowed us to understand when codes could be combined into a theme. We also used network visualizations, which helped us understand how different aspects of legitimacy may relate to one another on a broader scale (see Multimedia Appendix 2 for an example of one of these visualizations). This process allowed reviewer SH to maintain a differentiated perspective on each discipline and acted as a memory aid for the reviewer given the volume of the literature. SH then wrote summaries of the approach to legitimacy within each discipline, which were discussed with RW. SH coded the remaining articles using Excel (Microsoft Corporation) while editing the disciplinary summaries as necessary. Multimedia Appendix 1 provides an example.

SH developed a synthesizing argument that could connect constructs of legitimacy across all 3 disciplines. SH tested the fit of different conceptualizations with the disciplinary summaries and then explored the development of these metaphors with RW. After SH had read 90% (87/97) of the articles, no further edits or additions to the disciplinary summaries or synthesizing argument were necessary, likely indicating data saturation.

## Results

### Organization and Management Studies

#### What Does Legitimacy Mean?

The basis of legitimacy epistemology in OMS can be traced to the highly influential paper by Suchman [14]. His definition of legitimacy still forms the basis of most subsequent theorizing in this field: “Legitimacy is a generalized perception or assumption that the actions of an entity are desirable, proper, or appropriate within some socially constructed system of norms, values, beliefs, and definitions” [14].

This definition builds from and expands on Weber’s [127] understanding of legitimate authority, among other conceptualizations [41], focusing on the application of Weberian legitimacy concepts such as “tradition, affectual attitudes, value, and legality” [127] within a “socially constructed system of norms, values, beliefs, and definitions” [14]. Bitektine and Haack [128] further expanded on the Weberian connection to Suchman [14] with a multilevel legitimacy theory that differentiates among legitimacy judgments at the individual and collective levels [42]. Because of strong theoretical cohesion in this discipline, authors draw generously from these theoretical traditions, and most legitimacy subtypes are variations of normative legitimacy. Normative legitimacy deals with the norms, systems, and underlying assumptions that result in an institution, technology, or system being considered legitimate, and is generated through systems and social processes that may not be made explicit until there is a legitimacy crisis [41].

This definition is notable for its understanding of legitimacy as a perception of actions based on socially constructed norms and implicit conceptualization of legitimacy as a taken-for-granted state. However, perhaps more important is what is left out: because legitimacy is “generalized,” it is not dependent on materialities or physical space, nor does the definition of legitimacy change specific to relationships among institutions or individuals. Our critical interpretive analysis shows that these kinds of assumptions seem to help determine the lines of reasoning for most OMS legitimacy scholars included in this review.

Our analysis shows that OMS legitimacy studies constitute a cohesive body of literature in which theoretical contributions are made incrementally. For instance, most authors outline exactly how they are adding to the existing literature (usually based on the definition by Suchman [14]) in a specific way. For instance, articles often divide legitimacy into ever more granular categories such as “network legitimacy” [43] and “actional legitimacy” [44] or focus on a particular aspect of the definition by Suchman [14] to explore in more depth, such as “normative appropriateness” [129]. Therefore it is easy to trace the lineage of theory. However, because every article adheres to the same framework, there is less diversity of thought. In light of this, a division could be made between technology literature and health care literature. Technology literature, usually with an entrepreneurship bent, tends to focus on audience perception and innovation framing through discourse, by teaching entrepreneurs how to appeal through discourse to different kinds of funding audiences [45]. For example, entrepreneurs seeking crowd-based funding are advised to make “contribution claims” emphasizing identity and added value to the funding community, rather than institutional ties or the significance of potential scientific or social advancements that may legitimate the entrepreneur to other audiences [45]. Health care literature focuses mainly on institutional legitimacy, for instance, by examining the negotiations involved in academic health center mergers [46] or the role of physician executives in institutional contexts [47]. For example, Kitchener [46] emphasizes that even as new sources of legitimacy for an academic health center became necessary in the wake of both a merger and a changing environment, the aim was to create an “aura” of legitimacy within a new institutional logic; in other words, even though the source of legitimacy changed, it was still defined through institutions.

#### How Is Legitimacy Produced?

On the basis of the literature reviewed for this study, conceptualizations of legitimacy production in OMS fall into 3 categories. Legitimacy-as-process, or legitimation, is “an interactive process of social construction” [25], often with clearly delineated steps toward generating legitimacy [48,130]. Articles using this conceptualization tend to track change over time [13,49,50], for instance, by examining the legitimation journey of a single innovation over decades [50]. Legitimacy as perception or “a social judgment, an evaluation, a socio-cognitive construction” [25] is used most frequently in relation to audiences or the question “legitimate to whom?” usually when examining innovations within larger organizational contexts [45,51,52]. Finally, a legitimacy-as-property conceptualization, in which legitimacy is viewed as an asset to be gained, increased, or lost [25], emphasizes how an entity gains legitimacy rather than differences between legitimate or illegitimate entities; this conceptualization appears to play a smaller role in more recent work. Legitimacy as a process and legitimacy as a perception are often used simultaneously within the same paper; there is an acknowledgment that both perspectives can offer valuable insights. For instance, McKnight and Zietsma [53] describe the process through which new ventures can achieve “optimal distinctiveness” (a perception) through framing and collaboration strategies (a process). Similarly, Garud et al [54] discuss entrepreneurial storytelling as a legitimation process that relies on audience perceptions, thereby promoting an understanding of audience perceptions as part of analyses of different narrative techniques, which are framed as processes of legitimation [53].

#### How Is Legitimacy Used?

Within the literature reviewed, we noted that legitimacy is often assumed in OMS to be necessary for resource acquisition and is mostly relevant in an institutional setting. Because resource acquisition is the *raison d'être* of business in capitalism, legitimacy is considered essential. All literature included in our review aims to explain legitimation journeys or to advise institutions and entrepreneurs on how to move toward legitimacy. The stated goal is often to advance legitimacy theory within OMS, rather than explore empirical evidence beyond specific case studies.

Our analysis shows that nearly all legitimacy problems in OMS are framed as problems of categorization: either an institution or innovation has been miscategorized by its intended audience, or it has not yet been categorized [51]. Because of this, there are several articles that deal with the paradox of legitimacy for disruptive innovations: the innovation must conform to a category to be seen as legitimate, but it will not be seen as necessary unless it also distinguishes itself within the category [45,53,54]. Articles often describe how entrepreneurs can strike this balance or analyze how an older innovation navigated the paradox. For instance, Ruef and Scott [41] produced a multidimensional model of legitimacy designed to help hospitals use knowledge of legitimacy norms and categorization to “attract managerial legitimacy.” Other authors provide articles that, while academic, can also be interpreted as how-to guides for entrepreneurs attempting to formulate narratives for different audiences. Articles in this category posit that entrepreneurs must fit the narrative to the correct category of both the legitimacy problem [54] and the audience’s perspective [45].

Articles discussing specific innovations often explore problems of categorization through “institutional logics” [45,46,55] or the forms normative legitimacy may take within a particular institution. These articles acknowledge that different institutions have different norms, and therefore, entrepreneurs and innovators must adapt their storytelling to “fit” with the institution. Our analysis of the OMS literature showed that institutions that are taken for granted have no narrative flexibility. This means that any changes made not only require buy-in from professionals but also immediate practical implementation of changes (such as shifting patient education responsibilities from physicians to nurses) to avoid becoming mired in default institutional processes [56]. However, an entrepreneurial venture wrestling with liability of newness [53] has nearly endless opportunities for narrative reframing and limited risk of stagnation in nonexistent institutional processes [45].

#### Summary

In short, OMS seems to heavily theorize granular definitions of legitimacy, derived mostly from normative legitimacy. Most articles describe how organizations can move from illegitimate to legitimate status. Legitimacy must be negotiated at multiple levels and can be conceptualized as a process, perception, and asset. On the basis of our initial analysis, legitimacy in OMS rests on taken-for-grantedness and categorization according to institutional logics; it is hampered by liability of newness [51] and difficulties fitting into an institutional logic or other category.

We now move on from the critical interpretation phase of CIS methodology and toward synthesis. We develop synthetic constructs to metaphorically unite major themes within each discipline. While the OMS line of reasoning is somewhat homogeneous, our interpretive analysis shows that there is acknowledgment that different institutions use different systems of thought, or institutional logics, to judge and negotiate legitimacy. Stakeholders must navigate these different systems by fitting themselves into clear categories. However, the system by which legitimacy is judged and negotiated is rarely fully navigable to any one stakeholder.

In this way, navigating legitimacy in OMS is like using a highway system. The roads themselves (in this case, pathways to legitimacy) are clear and concrete but can only be used by particular categories of vehicles in particular locations, in the same way that, for instance, entrepreneurs must use particular discourses (ie, roads) to legitimate their invention to certain audiences (locations) [45,50,54]. The metaphor of the highway system allows us to capture OMS’ conceptualization of legitimacy as a rule-bound system of connections, driven by institutions. The highway system metaphor shows us that the OMS perspective on legitimacy is unusual for how fully theorized and comprehensive it is. However, in the same way that mapping a highway system alone does not describe everything that happens on the road, this metaphor also helps us articulate gaps in OMS theorization. OMS focuses on pathways to legitimacy, institutional involvement, and categorization; however, it can be difficult to explore the moments of legitimacy breakdown, unexpected contradictions within legitimacy journeys, and the influence of materialities on legitimacy within the concrete and bounded pathways that make up legitimacy conceptualization in OMS.

### Science and Technology Studies

#### What Does Legitimacy Mean?

Within the legitimacy literature reviewed, nearly all STS articles dealt with health care or science practices on the edge of scientific validity or public acceptance, usually by focusing on controversial technologies. Authors seemed to pay significant attention to the audience for these controversies. Innovations explored within STS also include changes in health care and scientific systems or discourses, for instance, the process of creating new institutions within an academic environment [57].

In this way, it seems that legitimacy in STS can only be discussed easily when it is in question. This may be linked to STS’s longstanding focus on controversies as strategic locations of research and the STS tendency to “stay with the trouble” [131]. Taken-for-grantedness may be considered such an integral part of legitimacy that it raises red flags in a discipline that is highly sensitive to unintended consequences.

However, in the STS literature reviewed, legitimacy itself is rarely the subject of an article. Our interpretive analysis shows that legitimacy is still undertheorized in STS, in that many articles do not reference other legitimacy literature or define the term. When legitimacy is explored in depth, authors tend to deal with it as a type of knowledge coproduced at the site of controversy between human and nonhuman actors. For instance, 2 articles examine the demarcation of boundaries in discourse about future-facing technologies; in these articles, narratives about nanotechnology and forensic science both impact and are impacted by developments in the technology [16,58]. For instance, Selin [16] demonstrates that even if a single vision of the future of nanotechnology is accepted by scientists over all others, competing visions may still have an impact on the direction of technological development via other avenues, such as governance and funding. We interpret this as evidence that legitimacy is coproduced between the narrative around new scientific disciplines and concrete innovations within those disciplines.

A consequence of the focus on disputed boundaries that we observed in the STS literature seems to be that STS scholars treat legitimacy as mutable. Boundary work [59] articles conceptualize legitimacy as a process that happens through negotiation within relationships among people and institutions. Boundary work deals with how people and institutions negotiate changing roles, limits, and responsibilities, often in relation to knowledge practices [59]. Within our review, most authors seem to use boundary work only as an explanatory framework for discursive strategies, but some articles include writing and practices as instances of boundary work [60]. For instance, Granja and Machado [58] describe how forensic scientists clearly demarcate their expertise and the limits of their role in the justice system through both discursive strategies and practical performances of accountability such as fastidious record keeping as a strategy to maintain their legitimacy. While perception can play a role in this process, we found no STS boundary work articles within the literature reviewed that consider legitimacy to be merely an asset. We interpreted this as possible evidence of the STS disinclination to consider any relationship or object to be fixed and a general disciplinary focus on processes of knowledge production.

#### How Is Legitimacy Produced?

We found that processes of legitimacy coproduction of new technologies in health care are described in relationships among a variety of human and nonhuman actors [28]. STS seems to resist explanations that pin legitimacy’s presence or absence on a single explanation or strategy. Instead, by examining the influence of nonhuman actors, STS encourages readers to embrace complexity in legitimacy relationships. For example, Barker [61] discusses how a drug marketing campaign legitimated a contested illness; McLevey [62] details how legitimacy goals for policy recommendations change how think tanks make knowledge; and Greco [63] uses the concept of “scientific ideology” to explore how the placebo effect threatens the legitimacy of scientific rationality in biomedicine. In these examples, the influence of unexpected nonhuman actors shapes legitimacy in a porous way; this influence adds complexity to legitimacy relationships. These examples thus turn the “expected” legitimacy relationship upside down. Barker shows that a treatment can make an illness legitimate (rather than an illness becoming legitimate and then treatment being developed). McLevey [62] demonstrates that knowledge may be made to legitimize policy recommendations (rather than knowledge first being created and then informing policy decisions). Finally, Greco [63] posits that a biomedical effect can threaten the legitimacy of biomedicine (rather than biomedicine creating biomedical effects that reinforce its legitimacy).

#### How Is Legitimacy Used?

Through our critical interpretive analysis, legitimacy in STS appears to be yet another “two-faced Janus” [28], in that it is coproduced by both taken-for-granted processes and unexpected assemblages, which may appear contradictory. For instance, Brown and Michael [64] note that the institutional mechanisms for validating scientific discovery play a part in legitimizing medical use of pig organs. However, the authors also find that these taken-for-granted institutional processes are not enough to legitimate transspecies transplantation. Rather, porcine donor organs are legitimated in their interpretation through an unexpected assemblage of institutional processes and scientific and cultural judgments about pigs. While anatomical similarities between humans’ and pigs’ organs exist from a scientific perspective, human-pig differences legitimate porcine donor organs (over, for instance, monkey organs) from a cultural perspective [64]. These apparent contradictions do not threaten the legitimacy of porcine donor organs; instead, the authors interpret these contradictions as necessary to establish legitimacy for a controversial innovation in medicine.

While most articles in the reviewed STS literature use the concept of legitimacy to explore complexity in relationships among technologies, ideas, people, and institutions, a small subset of the literature consists of discourse analyses focusing on language as the only vehicle of legitimation [4,62]. In these articles, there is no room for other means of legitimation, such as nondiscursive practices, language-free materialities, or physical space. In addition, policy-oriented articles about discourse-based legitimation strategies tend to view these strategies as mutually exclusive: “legitimacy can only be achieved if complexity is reduced via language” [65]. This appears to be a radical departure from the assembled view of legitimation present in most STS articles, in that legitimacy is produced by specific arguments designed to convince one party that another is legitimate, rather than *through* the complexity of negotiation among people, technologies, ideas, and institutions. However, both STS subgroups use legitimacy as a relational concept, whether relationships are implied through arguments or complex negotiations.

#### Summary

Our analysis demonstrates that STS approaches legitimacy through controversy and contestation. Because of its empirical orientation and wide variety, the way legitimacy is approached in STS cannot be viewed as theoretically cohesive. We found that STS explores legitimacy as an artifact of knowledge coproduction, centering nonhuman actors and complex legitimacy processes through discourse, practices, or both. In one conceptualization, legitimacy is viewed as a constant and complex negotiation among actors and their contexts. However, discourse analyses often view the complexity of these relationships as a problem for legitimacy rather than an integral aspect of the concept. Despite this divergence, nearly all STS literature reviewed seems to assume that legitimacy is relational and includes nonhuman actors, contexts, and practices of knowledge production.

Moving toward our synthetic constructs, we posit that in STS, the coproduction of legitimacy through both taken-for-granted processes and unexpected assemblages functions such as the mutable infrastructure of a computer operating system. The hardware of the computer does not change (much), even though the overall capacity of the computer relies on constant updates of existing software. New operating systems build on what came before while adding something new to stay relevant. Similarly, the legitimacy of technologies in health care is viewed predominantly as being built on underlying infrastructures, such as evidence-based medicine (EBM) and laboratory protocols. However, it is also considered to be shaped by unexpected influences, such as the coexistence of traditional medicines with EBM standards [66] and popular conceptualizations of new technologies that do not align with the use of those technologies by experts [58]. Furthermore, legitimization of a technology may have unexpected consequences, such as a medicine legitimizing an illness [61]. Legitimacy, according to STS scholars, must be constantly negotiated among nonhuman actors through an iterative process that is most visible at the boundaries. Through the metaphor of computer operating systems, STS emphasizes a dualistic perspective of legitimacy. Legitimacy in STS depends on the mutability and unexpected creativity of practices, actors, and context (ie, software); simultaneously, this “software” of legitimacy must be built on top of preexisting systems that are much more rarely changed in substantial ways (ie, hardware). Without these preexisting systems, the knowledge production practices delineated by STS scholars have nowhere to operate.

### Medical Anthropology and Sociology

#### What Is the Meaning of Legitimacy?

On the basis of our critical interpretation of the MAS literature under review, we found that MAS tends to center relationships among people within larger systems and physical spaces, rather than the nature of the systems or institutions themselves. Similar to STS, the MAS literature reviewed usually discusses controversies rather than strategies of legitimation.

Because few articles use concrete definitions of legitimacy or conceptualizations that reference any other body of literature, even if they discuss legitimacy issues in depth using empirical evidence, we found legitimacy to be undertheorized within MAS as a discipline. Several articles use terms such as “medical legitimacy” [6] or “narratives of legitimation” without definitions [67]. Some define legitimacy only in relation to another concept [12,68,69]. More than any other discipline, MAS seems to veer toward legitimacy definitions that are limited by subject and narrow [59] in scope: for instance, “being legitimately on sick leave requires both a certified illness and the inability to perform one’s work as a result of that illness” [70]. In our interpretation, this appears to allow for more empirical grounding but prevents conversation with other literature about legitimacy as a larger concept. Within the MAS literature reviewed, legitimacy is always relative, in that there is no universal legitimacy that works across all contexts.

However, there is still some cohesion visible among a subset of articles in the discipline. Several authors frame legitimacy as a form of “symbolic capital” in the Bourdieuvian sense [69,71,132] that can be used for resource acquisition. These articles and others also use boundary work [59] to describe the legitimacy of professional roles as a negotiation of boundaries mostly through discourse [72]. In combination with the disciplinary tendency to focus on the disenfranchised, these framings paint legitimacy as a power issue [37].

The literature reviewed seems to prioritize nuanced accounts of both social processes in contested situations and the spaces and materialities that shape the contestation of a technology. For instance, in an exploration of medical quackery in South Africa, Hornberger [68] shows that even when using medically useless technologies, providers of these technologies can construct legitimacy for their patients because of contextual elements, such as lower education levels, local beliefs, and problems accessing proven medical treatments. Spaces, materialities, and relativity appear to be especially important in articles about disconnects between policy and practice in professional settings [73]. For example, Perrotta and Geampana [7] explore how in vitro fertilization (IVF) professionals navigate the disconnect between the standards of EBM and the enthusiasm of patients for the latest technologies. In another account of IVF, Barnreuther [84] notes that because the research took place in a physician private home in India, as opposed to an institutional setting in a Western country, the resulting technical success was deemed illegitimate and went unrecognized in both medicine and research for decades. We find that these kinds of nuanced accounts present a deeper and more empirically grounded understanding of the meaning of legitimacy in context, despite a lack of theorization.

#### How Is Legitimacy Produced?

Our critical interpretation of the MAS literature emphasizes the intertwined nature of practices, discourses, social processes, and materialities in the production of legitimacy. Authors often seem to assume these are impossible to separate from each other without losing meaning. For instance, in a study of the use of restraint in a hospital psychiatry ward, McKeown et al [74] examine how boundaries of space (ie, office or patient area), identity (ie, health care professional or patient), and diagnosis work together to legitimize or delegitimize the use of force in context. In this way, legitimacy is defined, not merely influenced, by the environment in which it is generated [75].

When legitimacy is conceptualized as a power problem, categorization seems to solidify the standing of already-legitimate actors. Meanwhile, power appears to become less accessible for the illegitimate when professional boundaries are unclear [69,76-78] or impermeable to outsiders [8,79,80]. Common MAS topics include contested illnesses and attempts of patients to receive treatment [70,80,81] and role negotiations of nonmedical practitioners within the medical establishment [69,79,82-84]. In both cases, power imbalance is usually framed as the primary obstacle to legitimacy, which is necessary for a more egalitarian redistribution of power. Although some authors argue it is possible to have some power without legitimacy [72,82,84], the physical location of institutions, people, and technologies still defines flows of resources, manifesting in, for instance, differential treatment of patients at a rural hospital that would be unlikely at an urban hospital due to differences in hospital conditions rather than patient deservingness [85]. On the basis of these examples, our interpretation is that MAS and OMS have some overlap in this area in that both disciplines seem to approach legitimacy as an issue of access to resources.

#### How Is Legitimacy Used?

MAS literature reviewed often examines how legitimacy can flow through the taken-for-granted artifacts of professionalism. These artifacts include prescriptions [78] and complicated-looking fake medical machines [68]. Because the object itself is imbued with legitimacy through specific qualities in the professional context (eg, an appearance of being “official” or “high tech”), artifacts in these articles seem to reinforce the legitimacy of the pharmacist writing the prescription or the practitioner using the machine. Material artifacts may also include larger systems such as old IT infrastructures or preexisting contracts that impede desired change within institutions [73], implicitly reinforcing the legitimacy of institutional systems. While these materialities impact potential changes in legitimacy, they are usually not the subject of debate in the MAS literature. Therefore, it seems that the legitimacy of the prescription-writing pharmacist may be contested, but the prescription itself usually is not [78].

#### Summary

Our critical interpretation of the MAS literature is that legitimacy in this discipline is a relative and context-dependent concept. Legitimacy is often understood to be intertwined with power relations, but authors disagree about the nature of this connection and its implications. Finally, MAS articles center sites of controversy in which the subject has already been deemed illegitimate by focusing on space, materialities, and relationships. MAS authors appear to use these lenses to question “default” narratives of legitimacy in favor of exploring how and why a technology, system, group, or experience came to be viewed as illegitimate.

Here we develop our final disciplinary synthetic construct. Our interpretive analysis found that in MAS, legitimacy is physical, material, and always relative. Therefore, we employ the metaphor of a building’s plumbing: each part of the system, from the pipes to the faucets, performs a different function, which defines both their physical form and location. No two buildings can have identical plumbing systems, because no two buildings, even if constructed similarly, are situated in exactly the same location—thus, they must connect to the local environment in different ways. In much the same way, the materialities of health and technology, and how these materialities interact with spaces and actors, are considered to define the legitimacy of health and technology in MAS. A “quack” technology reads as legitimate instead of “fake” because of both its physical form and the spatial context it inhabits [68]; an innovation such as IVF in the “wrong” space may never be recognized as innovation at all [84]. In both cases, if we understand legitimacy to be an infrastructure that is dependent on both space and materialities, we can better connect the different underlying systems and elements that comprise legitimacy for health technologies. A sink connected to a sewer line requires multiple specific parts, arranged in a particular way, running through a particular location, to function. In the cases noted earlier, materials, the spaces in which they are developed, and the spaces in which they are used must be arranged in particular ways for technologies to be considered legitimate, whether the audience is the international scientific establishment [84] or residents of a rural South African community [68]. The metaphor of a plumbing system emphasizes the categorical importance of context and physical materials in the construction of legitimacy: in MAS, legitimacy cannot exist without these. Therefore, this metaphor also helps us understand why MAS generally avoids comprehensive definitions or theorization of legitimacy; other than a few basic building blocks, systems of legitimacy for MAS are essentially viewed as nontransferrable between contexts.

### Synthesizing Argument: Legitimacy as Social Infrastructure

Legitimacy is omnipresent and important within all 3 disciplines. However, it is often undertheorized and the subject of many implicit assumptions across MAS and STS literature. In OMS, conversely, increasingly detailed theorization results in the proliferation of “subtypes” of legitimacy with limited empirical support (see Table 1 for more details). Our synthesizing argument allows us to bring these different disciplinary traditions into conversation with each other through a new conceptual framework for legitimacy.

**Figure 2:**
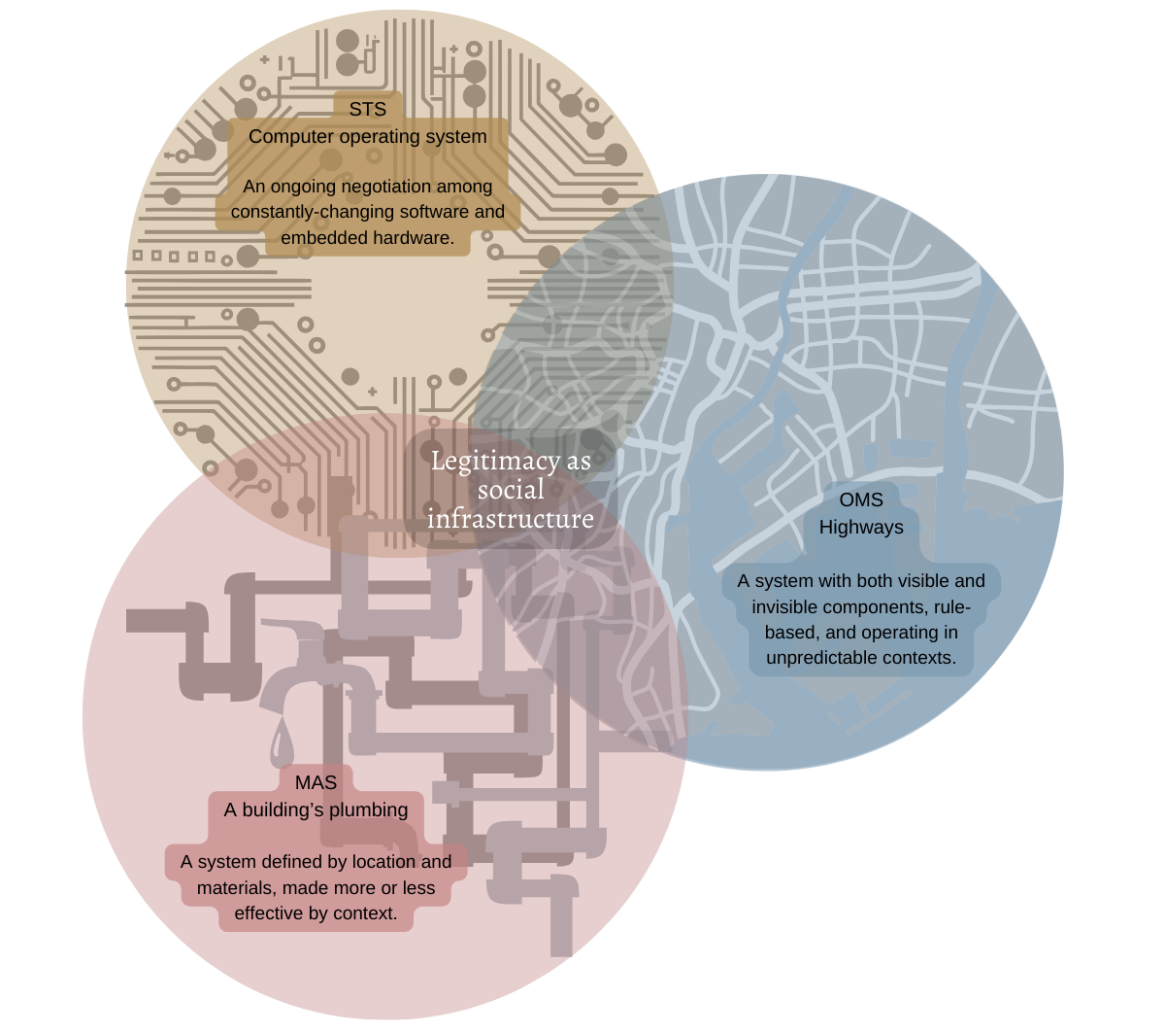
Disciplinary metaphors. MAS: medical anthropology and sociology; OMS: organization and management studies; STS: science and technology studies.

Using the 3 disciplinary metaphors we developed, we propose that legitimacy can be conceptualized as a social infrastructure, based on the criteria for an infrastructure by Star and Ruhleder [133]. If we combine the metaphors of a highway system (OMS), computer operating system (STS), and plumbing system (MAS), we begin to see the building blocks of an infrastructure that can support an entire city (Figure 2). Each metaphor shows a particular element of city infrastructure (ie, understanding of legitimacy) clearly. However, similar to a layered map of a city’s interconnecting infrastructures, we gain a fuller picture of how legitimacy works in health and technology when we layer the metaphors to view their intersections and divergences. Knowing how computer systems monitor traffic and manage stoplights helps us better understand both computer systems and highways; similarly, an STS understanding of how unexpected assemblages may work with taken-for-granted processes can help us better understand how these unexpected assemblages may impact theory about taken-for-granted processes in practice within OMS. Therefore, such a layered map allows for a richer conceptualization in which there is room for different approaches to legitimacy without denying the validity of any single conceptualization.

**Table 1:** Disciplinary characteristics: legitimacy in organization and management studies (OMS), science and technology studies (STS), and medical anthropology and sociology (MAS).

	OMS	STS	MAS
Theoretical foundations	Major focus on theory, granular definitions of legitimacy common; normative legitimacy most prevalent	Some theoretical exploration but limited theoretical cohesion	Limited theoretical exploration, reliance on granular definitions based on empirical evidence
Conceptualized as	A process (of negotiation); perception; or asset, and sometimes as several of these at once.	A product of knowledge coproduction; a negotiation among human and nonhuman actors, contexts, and epistemic cultures	A relative and context-dependent concept intertwined with power relationships
Purpose of literature	Describe how organizations can move from illegitimate to legitimate status	Investigate instances of controversy and contestation	Explore how and why a subject came to be viewed as illegitimate
Most important related concepts	Taken-for-grantedness; categorization and standardization	Relationships, taken-for-grantedness, and unexpected assemblages	Materialities, relationships, and space

Similar to a physical infrastructure, legitimacy can be understood as a binding fabric that makes possible certain types of relationships among people, institutions, and technologies. In our conceptualization, legitimacy is an inherently relational concept: it can only exist between two or more entities, rather than within the perceptions of a single person or institution. The notion of social infrastructure makes space for legitimacy to exist outside of human judgments, without devaluing the importance of perception in the production of legitimacy. Just as infrastructures consist of both materialities and abstract systems (eg, roads are made of concrete, but their use is facilitated by traffic laws), social infrastructure melds semiotic, material, and normative aspects of legitimacy and explores how they might work together. Infrastructure thus allows for the integration of disparate and seemingly contradictory aspects of making legitimacy. While Star [134] has emphasized that infrastructure is inherently a “relational property,” we use the word “social” to focus on how legitimacy supports specific types of relationships among people, institutions, and technologies; in particular, how these relationships are shaped by implicit and intangible norms and values [135]. For instance, understanding the nuances and norms of pharmacists’ relationships with both physicians and health care institutions reveals why a simple policy change allowing pharmacists to prescribe medication legally is not enough to legitimize the practice [78]. This moves us away from conversations about legitimacy as a conduit for resources and toward more clarity about how legitimacy acts as a binding fabric within social relationships.

Descriptions of legitimacy across all 3 disciplines fit the criteria for an infrastructure by Star and Ruhleder [133]. For instance, legitimacy is usually only visible “upon breakdown” [133]. Contestation and controversy are the only sites of legitimacy exploration in MAS and STS; in OMS, legitimacy is discussed in terms of desired change in legitimacy status (ie, moving from illegitimacy to legitimacy), and contestation is captured explicitly through concepts such as “liability of newness” [53,54]. In addition, maintenance of this social infrastructure happens through continued adherence to legitimizing protocols and norms within relationships; deviations can result in a crisis of legitimacy. In this sense, legitimacy is characterized by a strong fit with predefined categories and “standards” [133] in many articles [45,53,65,85], even when legitimacy itself is not defined. In our conceptualization of legitimacy, norms and values make up the “installed base” into which legitimacy is embedded [133] and are responsible for legitimacy’s taken-for-granted nature.

Legitimacy also acts similar to infrastructure, in that it can be used to invisibly support the work of organizations, technologies, or people [55,86,133], often by allowing for acquisition of resources or power [69,87]. In fact, values and norms are often so deeply entrenched and invisible that even the infrastructure supporting them (ie, legitimacy) becomes taken-for-granted and invisible itself. This combination can make it extremely difficult for any contradictory experiences or knowledge to find expression within a society’s taken-for-granted, unquestioned truths [85,88,89,132]. Therefore, it is understandable why the moments of crisis when legitimacy is “visible upon breakdown” usually occur when norms and values clash with each other. For example, in the exploration of IVF by Perotta and Geampana [7], new reproductive technologies are a site of legitimacy crisis because they are heavily desired by patients but cannot adhere to standards of EBM. In this situation, physicians must navigate among the medical value of providing patients with the care they seek, the medical value of providing evidence-based care, and the medical norm of adhering to preset medical standards that may not have kept up with available care options.

Finally, the origins of legitimacy parallel those of any other infrastructure. Legitimacy is built on existing social systems: it “wrestles with the inertia of the installed base” [133] and borrows both strengths and weaknesses from that base, resulting in legitimation by once-functional mechanisms that are now relics. For instance, Sheard et al [73] show how desired changes in practices in a hospital setting are stalled by existing social, technical, and bureaucratic systems and entrenched social practices, despite a lack of effectiveness of these entrenched systems and a desire for change among the people acting within them. Overcoming this systemic inertia requires the integration of new practices and systems into the installed base, regardless of the current utility of that base; attempts to completely overhaul entrenched institutional practices or technologies without attention to the installed base rarely succeed.

In all 3 metaphors, infrastructure serves as a system through which different elements that contribute to legitimacy can negotiate with one another. A social infrastructure conceptualization reflects the nature and parameters of these negotiations. Thus this lens allows us to see where different perspectives on legitimacy could provide a more holistic picture of how legitimacy works in practice, encouraging a collaborative approach to conflicting views on legitimacy.

We hope this conceptualization can provide a useful lens to better understand how legitimacy is produced and how it works among institutions, technologies, and people. We aim to move the conversation toward a more holistic perspective on embedding technologies in health care that goes beyond models based primarily on judgments and perceptions.

## Discussion

### Principal Findings

Our conceptualization of legitimacy as social infrastructure allows for deeper analysis and understanding of the meaning, production, and uses of legitimacy when embedding technologies in health care. Our review contributes to the OMS literature by bringing in outside perspectives. Cross-fertilization among the 3 disciplines reigns in the proliferation of legitimacy theory in OMS while providing a better connection with empirical research. STS and MAS literature benefit from grounding empirical exploration within a more cohesive theoretical framework. Our review also helps contextualize studies of legitimacy that are more limited in scope; for instance, while discourse analyses of legitimacy are useful, this review demonstrates that discourse comprises just one aspect of legitimacy production [67,90,91]. By allowing for many definitions of legitimacy, the notion of legitimacy as social infrastructure is adaptable enough to produce meaning across disciplines while providing ways to explore relational, material, and semiotic aspects. By providing a framework through which to explore many conceptualizations and understandings of legitimacy, we hope LSI can facilitate fruitful cross-disciplinary collaborations of legitimacy researchers. Specifically, LSI allows different conceptualizations to coexist without competing with one another, providing a framework in which researchers can build on the work of other disciplines without having to disregard the traditions of their own discipline. In addition, this framework provides value to acceptance, adoption, and implementation researchers and other stakeholders interested in better explaining how technologies are embedded in health care. Through an interdisciplinary understanding of legitimacy and a focus on relationships and moments of breakdown, LSI provides a mechanism to study technology embedding both contextually and over time. In this way, LSI produces a more comprehensive understanding of how the interconnected social, medical, environmental, and institutional elements surrounding a technology may interact in ways that impact legitimacy for a given technology, and thus, whether a technology will become embedded in health care.

### Embedding Beyond Current Frameworks

Legitimacy as social infrastructure provides an alternative to current models describing how technologies are embedded in health care. We can use this conceptualization to account for gaps between policy intentions and practices when embedding technologies. Social infrastructure produces a more comprehensive understanding of the issues underpinning technology adoption than frameworks that hinge on trust [136,137], transparency [4,137], and acceptability [19], especially when those frameworks are based on behavioral intention [20,21,138]. Articles included in this review have shown that the complexities of embedding technologies in health care often clash with the best-intentioned policy choices because these frameworks generally do not account for the full scope of legitimizing processes within health care delivery systems. This is particularly evident in articles that focus on contested illnesses [80,89,92] and professional boundary work negotiations in light of new policies or collaborations in health care [47,56,77,78,93,94]. For example, Weiss and Sutton [78] demonstrate that even though policy changes explicitly allow pharmacists to prescribe medication, actually doing so in practice involves a more complex negotiation of roles and hierarchies to legitimize pharmacist prescribing activities. The new prescribing policy, based on notions of trust and acceptability, is not enough. For patients whose contested illnesses do not meet EBM criteria [80], negotiations and individual relationships are essential to access any kind of medical care [95]. Here, EBM criteria designed to increase transparency and trust in medicine prevent patients with real symptoms from accessing necessary care.

The many technology acceptance and adoption frameworks available currently have difficulty explaining adequately how and why some technologies are embedded in health care but others are not; in other words, “the literature does not specify the conditions for full use to be achieved” [138]. Similar to legitimacy, these terms are not clearly defined across the literature [138]: “acceptance” and “acceptability” generally are defined in terms of perceptions of potential users [138,139] and separate from both “adoption” and “appropriation,” which deal with the processes and practices of using the technology [138]. Other models aim to identify factors contributing to acceptance of new technologies. TAM is the most well-known example; it has been adapted and added to by numerous authors since its inception [20,21,138,140]. However, similar to most adoption and acceptance frameworks, TAM is based on behavioral intention, which has limited utility in predicting actual behavior [141,142] and does not generally attempt to integrate other systemic or relational aspects of technology acceptance. Within this review, Reay et al [56] demonstrated that gaining discursive buy-in, the focus of behavioral intention models of technology adoption, “is not enough” to legitimize new practices in a clinical setting without action and interpersonal, structural support.

Other frameworks have attempted to integrate sociocultural aspects of technology acceptance, specifically in low- and middle-income countries, although acceptance still generally focuses on individual users rather than systemic or relational factors [143]. More unusually, the nonadoption, abandonment, scale-up, spread, and sustainability framework by Greenhalgh et al [22], which is explicitly directed at health technologies, attempts to fill gaps left by TAM and other models by focusing on nonadoption and abandonment of technology instead of acceptance. This framework incorporates norms and values to some extent but does not focus specifically on legitimacy and the impact of norms and values on technology development.

The legitimacy as social infrastructure framework (LSI framework) is different from these models: rather than starting with adoption and acceptance of technologies, our conceptualization focuses on the relationships and negotiations that result in adoption and acceptance or not. If we take legitimacy as a necessary element in technology adoption and acceptance, conceptualizing legitimacy as social infrastructure allows us to understand how adoption, acceptance, and implementation frameworks may work together to explain the process of embedding technologies in health care. For instance, rather than conducting acceptance, adoption, and implementation studies separately in a health technology development project, researchers could use the conceptualization of legitimacy as social infrastructure as a framework to iteratively explore the binding fabric of relationships and legitimacy narratives that make up the landscape in which a new technology is being developed and provide advice to decision makers based on these findings. This focus would also allow project stakeholders to contextualize the results of a traditional acceptability or adoption study to better support the end goal of successfully embedding a technology in health care. Doing so would move conversation beyond simple adoption and acceptance and toward a more holistic perspective about this embedding process based instead on an analysis of legitimacy.

### Focus on Relationships and Narratives

Momentum for embedding technologies in health care often fades due to unforeseen complexities [144] when moving technologies from laboratories to real-world environments. Particularly for mobile health technologies in which social and relational elements play a heightened role, the LSI framework could prove extremely useful in both anticipating and navigating complexity in the real world by describing changing relationships and narratives. Legitimacy as social infrastructure could provide a better blueprint that takes contextual factors into account earlier and avoids some of the acceptance and adoption bottlenecks that develop in linear models.

The struggles and unintended consequences of embedding technology in practice have been well-documented in STS and OMS [58,61,93,96]. The LSI framework conceptualizes how these documented struggles impact legitimacy and, thus, technology adoption and acceptance. STS understands new technologies (including those designed for health care) to be both directional and open-ended [23]. Technologies are embodied with certain scripts and ideas, produced both through the development process and purposely in the minds of developers and other stakeholders, impacting how they can be used and when. However, the scripts managing this directionality never completely determine how a technology is used in practice [23,145]. Integrating technologies into real-world environments necessarily involves tinkering [22,23,144] by users, developers, and other stakeholders, often contradicting a technology’s directionality entirely. For instance, as Perrotta and Geampana [7] demonstrate, new medical technology may be used in ways that contradict its original EBM-derived script, given a particular set of circumstances generated by patients, economics, and the pace of technology development. What happens next is a negotiation that determines how real-world context, practices, and technology scripts interact to generate a legitimacy relationship. Conceptualizing legitimacy as social infrastructure allows us to both understand the nature of these interactions and how they may or may not lead to technology adoption and acceptance in a health care setting. For instance, Sheard et al [73] discuss the complex systems and legitimacy relationships that impact the ability of care providers in the United Kingdom to act on patient feedback; this feedback is solicited in the first place to increase trust in and acceptability of the medical system. If we view patient feedback frameworks as a form of health care technology, legitimacy as social infrastructure helps us better understand and anticipate the relative importance of backgrounded relational and structural systems [73] that determined whether patient feedback was incorporated successfully in the health care institutional systems under study. An infrastructural understanding reveals how the 3 issues identified (ie, normative legitimacy, structural legitimacy, and organizational readiness [14,73]) work together to support or deter change in a complex organizational setting. A binding fabric of relationships ties all of these issues together and clarifies why the quality of certain relationships is more important than others. This focus also adds to understanding of the phenomenon of “institutional entrepreneurs.” Relationships likely play a role in how institutional entrepreneurs avoid being “confined by the status quo,” allowing them to change organizations from within despite legitimacy challenges for others [73].

### Future Research and Applications

Given the utility of this conceptualization in bringing together 3 very different disciplinary traditions around legitimacy, future research could expand on this work to incorporate perspectives from disciplines, such as ethics and law, that we could not include here. In addition, it is possible that legitimacy is usually not explicitly conceptualized in medicine and psychology due to an implicit discipline-wide understanding of the values that underpin the concept. In other words, norms and legitimacy in medicine and psychology may be so closely intertwined with the goal of providing care that legitimacy may not be contested or questioned from these disciplinary perspectives. These kinds of taken-for-granted assumptions make it important to explore the origins, production, and meanings of legitimacy more thoroughly in the psychology and medical fields. Our conceptualization could allow for such investigation in fields in which the language describing legitimacy is either absent or perceived to be self-evident.

Potential applications of legitimacy as social infrastructure in practice have a wide range thanks to the flexibility and adaptability of the conceptualization. For example, to manage risks appropriately, regulators and others must have knowledge and understanding of the subject of regulation, in this case, health care technologies. This includes not only technical knowledge, but epistemological understanding of what is seen to constitute valid forms of knowledge, what is being left out, and “system knowledge of the context regulation is operating in” [146]. By approaching the embedding of technologies in health care as a result of knowledge coproduction, the notion of legitimacy as social infrastructure can provide deeper understandings of the systems and context of health technologies. It can then help regulators differentiate valid from invalid forms of knowledge in context. Regulators also experience legitimacy concerns themselves, in that regulation must be legitimate to function [9,147]. An understanding of legitimacy as social infrastructure could provide regulators with clarity about the practices, decisions, and structures that may impact their own legitimacy. For instance, knowing more about how their relationships with various stakeholders contribute to their legitimacy could help regulators take stakeholders’ viewpoints into account at the most impactful moments and more appropriately narrate their decisions for different audience needs.

### Limitations

Given the broad focus of our topic “legitimacy in health and technology,” we included a heterogenous collection of empirical topics within each discipline. While this allowed for a broad discussion of legitimacy in health and technology, we did not produce one overarching definition of legitimacy; rather, we developed a conceptualization that can be used as a framework with the diversity of definitions already available. Similarly, the disciplinary boundaries are somewhat “fuzzy” [24]: OMS is a subdiscipline of sociology, STS overlaps with both MAS and OMS, and MAS is a combination of 2 disciplines with similar approaches. We found these disciplinary constructs useful because, despite their overlap, they highlight substantial differences among social science approaches to legitimacy.

In addition to the legal and ethical boundaries previously described, in this review, we did not engage with several other topics related to the legitimacy of technologies in health care. While the word “legitimacy” is used frequently in medicine and health economics, very few articles in these fields define the concept or explore it in any depth. Rather, the vast majority use the word uncritically within a surface explanation of the importance of a problem, either by claiming without evidence that legitimacy is an asset held by organizations, technologies, or people [148-150] or by mentioning that the legitimacy of a technology is under threat but without fully discussing why or how [151,152]. When legitimacy is defined, it is often reduced to a series of related concepts, such as “expertise, reciprocity, and trustworthiness,” without discussing how these interact to create legitimacy [153], although there are a few exceptions [9]. Due to the general lack of critical exploration, medicine and health economics were excluded as disciplines. This is an area that would benefit from further research using the LSI framework.

For similar reasons, we focused our search strategy within journals known to provide deep conceptualization of legitimacy (see Multimedia Appendix 1 for further details). Given the abstract and conceptual nature of our subject, a database search proved inadequate for finding all relevant articles; however, searching only within specific journals, despite rigorous selection, may have biased study findings. We feel the benefits of closely adhering to CIS protocol in this manner outweighed this issue due to our study’s interpretive goals and the abstract subject matter.

### Conclusions

The notion of legitimacy as social infrastructure provides a deeper understanding of how and why technologies are or are not embedded in health care. Social infrastructure focuses on the aspects of legitimacy related to norms, semiotics, materialities, and specific relationships among stakeholders and institutional contexts without excluding or privileging one form of knowledge production over the other. Through this conceptualization, the production and use of legitimacy can be studied ethnographically by following the social infrastructural networks and relationships that constitute and maintain legitimacy. By moving beyond judgment- and perception-based frameworks, legitimacy as social infrastructure approaches the problem of embedding technologies in health care through the lens of knowledge coproduction. The LSI framework enables empirical data collection and analysis that transcends disciplinary boundaries without negating the discrete lines of theoretical thought of any one discipline.

Given the interdisciplinary nature of health care policy and practice and the wide range of stakeholders involved, clearer and deeper communication around legitimacy could have far-reaching impacts. This could include new frameworks explaining technology adoption and acceptance, greater consensus about what makes a health care technology or policy decision legitimate, and strategic direction for decision makers working with disruptive health care technologies within entrenched systems.

## Appendix

Multimedia Appendix 1 Method details: selection of journals, disciplines, and articles.

Multimedia Appendix 2 Methods: disciplinary network visualization.

## Abbreviations

CIS: critical interpretive synthesis
EBM: evidence-based medicine
IVF: in vitro fertilization
MAS: medical anthropology and sociology
OMS: organization and management studies
STS: science and technology studies
TAM: Technology Acceptance Model
WoS: Web of Science
