# Supplementary material for: Legitimacy as Social Infrastructure: A Critical Interpretive Synthesis of the Literature on Legitimacy in Health and Technology
Source: JMIR Hum Factors. 2025 Mar 5;12:e48955. doi: 10.2196/48955 (PMC11923462; doi:10.2196/48955)
Supplement: Multimedia Appendix 1 [file humanfactors_v12i1e48955_app1.pdf]

Multimedia Appendix 1: Methods Details

Development of Search Protocol

To compile a list of relevant disciplines (and journals within those disciplines), the first author first ran an initial search using Google Scholar, Web of Science (WoS), and the EUR library database search tool, using the terms “legitimacy” “health” and “technology”. These criteria were then refined to include “artificial intelligence.” However, it became clear that regardless of how specific the search criteria became, the majority of records returned through a database search would not include deep conceptualizations of the term “legitimacy,” because this term is commonly used across many disciplines as an unproblematic descriptor of technologies, legal processes, narrative choices, medical decisions, policies, and institutions. This was true even for articles in which “legitimacy” could be found in the title, subtitle, or abstract.

Therefore, we switched to a search for journals within particular disciplines known to publish more in-depth explorations of concepts like legitimacy in the context of health and technology. We first searched for journals through databases, specifically WoS and Scimago, selecting journals based primarily on impact factor. However, this did not yield strong results in several disciplines, notably Organization, Management, Policy, and Anthropology (because we could not search for journals in these disciplines within WoS or Scimago that specifically cover health and technology), and Science & Technology Studies (which is not listed as a separate discipline within WoS or Scimago). Therefore, we explored other means of selecting journals for these disciplines, primarily by using expert knowledge compiled by libraries at universities strong in each field in the form of library-based disciplinary guides.

Selection of disciplines and journals

Journals from within each discipline were selected manually based on the following criteria, which were selected to yield the most results related to theorization of legitimacy, legitimacy-making, and scholarship related to healthcare and technology.

- Journals with significant legitimacy theorization and/or articles with a strong focus on legitimacy, health, and technology.
- Journals with significant topical relevance in Aims and Scope.
- Journals included in curated academic lists of top journals within a given discipline from respected institutions
- (Relatively) high 2019 Impact Factor, as listed on Web of Science’s Journal Citation Report or Scimago Journal and Country Rankings

Legitimacy research spans a wide variety of disciplines, and given the interdisciplinary nature of this review, it seemed appropriate to seek to include relevant journals from as wide a range as feasible. Therefore, an initial search for journals included the following disciplines. This review will include articles published in English or published in translation in an English-language journal after initial publication elsewhere.

Management and Organization studies

Journals were chosen based on top journals within Erasmus University Rotterdam (EUR) library guide to Management studies. The initial list included journals categorized as General, Organizational, and Strategy/Entrepreneurship. For the purposes of this review, Management & Organization Studies (MOS) includes general management literature, studies of entrepreneurship, organization theory, and more specific studies within management, such as Health Management, because these areas provide both the majority of conceptualizations of legitimacy and are most relevant to technology adoption in healthcare.

Health Policy and Management

Journals were chosen from top journals within EUR library guide to Health Policy and Management.

Science and Technology Studies (STS)

A list of journals was compiled through journal guides created by the University of British Columbia, Maastricht University, and University College London. The list edited for journal content (for instance, history of science journals were excluded for relevance). Journals known to the reviewers and likely to publish highly relevant articles were also included.

Psychology

The reviewer conducted the search protocol within three top Psychology journals based on impact factor (Scimago) and possible relevance of topic within psychology. Because there were few legitimacy results, the reviewer conducted the search across four more journals in possibly-relevant topic areas with high impact factors. This search also yielded limited results.

Sociology

Relevant sociology journals are included on separate categories in the WoS Journal Citation Report: *Sociology* and *Social Science, Biomedical.* These lists also include thematic journals that are not relevant

to this project (either thematically, or because they focus primarily on ethics or law). The reviewer was also unable to locate a curated list for Medical Sociology. Therefore, the top Sociology journals were selected by gathering the top 15 journals listed under WoS Journal Citation Report category *Sociology*. The reviewer then integrated this list with relevant (i.e., sociological) titles within the top 15 listed under WoS Journal Citation Report category *Social Sciences, Biomedical.* This allowed inclusion of journals specific to medicine and health sociology. Some journals were included on both lists. The reviewer then ran the search protocol on these journals.

Medical Anthropology

Medical anthropology is known to the reviewers to include theorization on legitimacy, health, and technology, and was therefore included despite being a sub-discipline within Anthropology is general. This journal list compiled from University of California, Berkeley library (Berkeley hosts one of the original medical anthropology programs and is widely respected in the field, with a specialized Anthropology library). In Anthropology, impact factor is not as relevant as topic area, especially because *Anthropology* and *Social Science: Biomedicine* impact factor rankings include a huge diversity of disciplines, of which Medical Anthropology is a small subset; therefore, relevant journals are more likely to be found on a curated list from a top medical anthropology department.

Medicine

The reviewer searched the Erasmus Medical Center Medical Library database for high-impact general medical journals and medical journals with a focus on technology (WoC, Scimago). The reviewer excluded journals that publish only about biomedical treatment, biology, chemistry/pharmacology. The Lancet was the only top medical journal which contained relevant articles that either pursued legitimacy as a subject of investigation or problematized legitimacy in any way.

Search Protocol

Within these lists, the reviewer then ran the following search protocol within each individual journal:

- Legitimacy (or legitim* if search tool accepted wildcards)
- Legitimacy AND technology (or technol* if wildcards accepted)
- Legitimacy AND technology AND health (or technol* if wildcards accepted)

- Legitimacy AND "artificial intelligence"
    - If the first two searches yielded very limited results, the reviewer did not proceed with the second two searches. If the journal's title contained "technology" or "health," searches did not include these terms.
- Remove publications with limited results for legitimacy
- The reviewer summarized the results of the journal search protocol within a matrix (for instance "some legitimacy results, no tech, no health") for each journal under consideration.

Exclusions and Combinations

As a result of this process, Medicine and Psychology were excluded as disciplines within the review. The disciplines provide valuable context for the importance of a review of legitimacy literature, but do not contain significant literature theorizing or problematizing legitimacy in a way that relates to health technology applications. The exception was the Lancet, which returned legitimacy results. In this case, the reviewer may include particularly relevant articles from the Lancet manually in the review. Because there were few journals within Medical Anthropology and some overlap with journals categorized within Sociology (notably, Critical Public Health and Social Science and Medicine), Medical Anthropology and Sociology were combined into one discipline.

Four disciplines were initially selected. However, after thorough review of the descriptions of the journals on the Health Policy & Management list, the most relevant journals were better categorized within Management & Organization Studies. Several journals that emerged in searches for STS and Sociology & Medical Anthropology journals also appeared on the Health Policy & Management list. Therefore, the reviewer decided to eliminate Health Policy & Management as a separate discipline.

Final Journal Selection

The top ten journals within the three remaining disciplines (Management & Organization Studies, Science & Technology Studies, and Sociology & Medical Anthropology) were selected based on the following factors, in descending order:

1) Relevance

a. Many results for legitimacy, preferably with strong emphasis on health and technology, or with strong legitimacy theory results.
b. Aims and Scope of the journal fits with the aims of the project (does not focus on excluded or irrelevant categories)

2) Presence on relevant academic lists (especially if included on several lists across disciplines)
3) Impact factor
4) Diversity within the discipline

Journals with similar Aims and Scope were kept on lists when these journals returned exceptionally strong results during the search protocol, or if the topic had particular relevance to AI/mHealth in skin cancer diagnosis. However, if search results were not especially promising, the reviewer prioritized diversity within the discipline. In these cases, the reviewer first selected the journal with the strongest results from the search protocol. If these results were also similar, the reviewer chose the journal with the highest impact factor.

There was some question about how to categorize Social Science and Medicine, which appeared on Health Policy & Management, STS, Sociology, and Medical Anthropology lists. It was eventually categorized within Sociology & Medical Anthropology through the following process:

- The reviewer sorted through the abstracts on the first page search results for both “legitimacy” and “legitimacy AND technology.” Articles were categorized as follows:
    - Psychology (3)
    - Anthropology (8)
    - Sociology, including discourse analysis (22)
    - Political Science/Policy (9)
    - Health Economics/Evaluation (2)
- Due to the prevalence of Sociology and Anthropology articles, Social Science and Medicine was categorized as a Sociology & Medical Anthropology journal.

In addition, specific articles known to the reviewer to be seminal works in the field of legitimacy (or in legitimacy, health, and technology) will be included, even if these articles do not appear in the journals under review.

Journals with similar Aims and Scope were included when these journals returned exceptionally strong results or were particularly topically relevant. Otherwise, we prioritized diversity within the discipline to ensure that we captured the full breadth of legitimacy conceptualizations. In these cases, the reviewer first selected the journal with the strongest results from the search protocol. If results were similar, the reviewer chose the journal with the highest impact factor. Two journals (Social Science & Medicine and Critical Public Health) were designated as interdisciplinary due to ambiguous journal aims straddling MAS and STS and high utilization by both MAS and STS scholars. Articles in these journals were included in MAS unless there was compelling evidence that they should be included in STS instead.

Search Strategy

With the help of a librarian, a Boolean search string was developed and run through Web of Science in April 2021. The primary reviewer conducted a secondary search in each individual journal to ensure all relevant results were included. Search was initially conducted with the help of W.M Bramer, who wrote the Boolean search string to find all results in Scopus. This string yielded 481 results.

Scopus Search String

(EXACTSRCTITLE ("Social Science And Medicine") OR EXACTSRCTITLE ("Anthropology Medicine") OR EXACTSRCTITLE ("Health Interdisciplinary Journal for the Social Study of Health Illness and Medicine") OR EXACTSRCTITLE (" Information Communication Society ") OR EXACTSRCTITLE ("Risk Analysis") OR EXACTSRCTITLE ("European Journal Of Public Health") OR EXACTSRCTITLE ("Research Policy") OR EXACTSRCTITLE ("Qualitative Health Research") OR EXACTSRCTITLE ("Journal Of Management Studies") OR EXACTSRCTITLE ("Organization Studies") OR EXACTSRCTITLE ("Health Care Management Review") OR EXACTSRCTITLE ("Social Studies Of Science") OR EXACTSRCTITLE ("Organization Science") OR EXACTSRCTITLE ("Journal Of Business Venturing") OR EXACTSRCTITLE ("Sociology Of Health Illness") OR EXACTSRCTITLE ("Critical Public Health") OR EXACTSRCTITLE ("Social Epistemology") OR EXACTSRCTITLE ("Medical Anthropology Quarterly") OR EXACTSRCTITLE ("Science As Culture") OR EXACTSRCTITLE ("Health Care Analysis") OR EXACTSRCTITLE ("Science Technology And Human Values") OR EXACTSRCTITLE ("Medical Anthropology Cross Cultural Studies In Health And Illness") OR EXACTSRCTITLE ("Administrative Science Quarterly") OR EXACTSRCTITLE ("Sociological Theory") OR EXACTSRCTITLE ("Strategic Organization") OR EXACTSRCTITLE ("Medical Anthropology") OR EXACTSRCTITLE ("Big Data And Society") OR EXACTSRCTITLE ("AI Society") OR

EXACTSRCTITLE ("Journal Of Responsible Innovation") OR EXACTSRCTITLE ("Strategic Entrepreneurship Journal")) AND (TITLE(legitim*) OR TITLE-ABS(legitim* W/10 (health* OR medic* OR technolog* OR AI OR artificial-intelligen* OR machine-learning OR innovat* OR model* OR theor* OR concept* OR framework* OR wellbeing OR well-being OR care OR illness OR cancer)))

Unfortunately, this string yielded results from excluded journals, and no results from three included journals. Ulrich's web showed that these journals should have been listed in both Scopus and Web of Science. As a result, the primary researcher conducted a second search of these missing journals in Web of Science (WoS) on February 2nd, 2021.

WoS search string

(SO=Social Science And Medicine OR SO=Anthropology Medicine OR SO=Health Interdisciplinary OR SO=Information Communication Society OR SO=Risk Analysis OR SO=European Journal Of Public Health OR SO=Research Policy OR SO=Qualitative Health Research OR SO=Journal Management Studies OR SO=Organization Studies OR SO=Health Care Management Review OR SO=Social Studies Science OR SO=Organization Science OR SO=Journal Business Venturing OR SO=Sociology Health Illness OR SO=Critical Public Health OR SO=Social Epistemology OR SO=Medical Anthropology Quarterly OR SO=Science As Culture OR SO=Health Care Analysis OR SO=Science Technology Human Values OR SO=Medical Anthropology OR SO=Administrative Science Quarterly OR SO=Sociological Theory OR SO=Strategic Organization OR SO=Big Data Society OR SO=AI Society OR SO=Journal Responsible Innovation OR SO=Strategic Entrepreneurship Journal) AND TI=legitim*

After discovering that searches of just the missing journals yielded many results in WoS than in Scopus, she tried searching for journals that had been covered by the Scopus search string and discovered that these also yielded more relevant results in WoS. Therefore, the primary researcher translated the search string from Scopus terminology to WoS terminology and ran the search in two parts in order to ensure both elements of the search string were captured equally, as WoS provides significantly less opportunity for detailed searches than Scopus. The WoS search generated more results than the Scopus search (nearly 900) but did not include any results from the excluded journals.

Because of these problematic results from the Boolean search strings, the primary researcher conducted two searches through all included journals individually, using the following search protocol:The primary researcher then chose to exclude the terms "model*," "theor*", "concept*", and "framework*" because excluding these terms yielded more results and fewer mistakes, and because the title search alone was likely to find the

majority of these larger theoretical articles. This exclusion reduced the number of records from Search 2 by about 300. The primary reviewer then conducted a final search through each included journal using a revised version of Search 2:

Search 2: journal name + legitim* + technolog* and/or health

There were 224 results for Search 1 and 639 results for Search 2. When combined in EndNote, there were 144 duplicate results.

Inclusions

Articles that contain:

- Evidence of legitimacy theorization in the abstract either explicitly, or through causal relationship language (such as “these practices generate legitimacy”) OR the abstract indicates through discussion of the subject at hand that legitimacy is problematized even if it is not the focus of the article.

AND

- The subject of the article deals with health, technology, or both.

If an article covers only health or technology but not both, the threshold for legitimacy theorization is higher, i.e. only articles with explicit reference to theory of legitimacy or which use causal language to describe a relationship with legitimacy.

Exclusions

In reviewing abstracts, criteria for exclusion were refined but not expanded. In cases where legitimacy was only used in the abstract’s recommendations section, or when legitimacy was used as an adjective or descriptor in a list of other descriptors (with no other mentions), the article was excluded.

- Abstracts without good evidence of theorization of legitimacy. Specifically, abstracts were excluded if any of the following were true.
    - The abstract focuses entirely on the legitimacy of specific policies (no theorization of the meaning of legitimacy itself)

  - The abstract uses legitim* interchangeably with another term (for instance, “reasonableness” or “acceptability” or “ethical”).
  - The abstract mentions legitimacy only in the recommendations section.
  - Legitim* only occurs once within a list of other adjectives or attributes.
  - Legitim* is clearly not a central subject of the article.
- Abstracts that use legitimacy purely in a legal sense (well-covered by other reviews, beyond the scope)
- The subject matter is less relevant (deals with neither health nor technology)

A second reviewer (Rik Wehrens) then reviewed 10% of the total articles at random (every 10th article when arranged in alphabetical order by author name). The resulting inclusion/exclusion list closely matched the original reviewer’s (Sydney Howe, SH) selection, with a total of seven disagreements. After reviewing each disagreement, the two reviewers were able to come to an agreement on every contested article based on the inclusion criteria. Because all of these disagreements were in the first half of the articles reviewed, it was determined that these were mostly a result of fuzzier inclusion criteria during the first part of the sorting process. As a result, SH went through the first half (up through author last name letter M) to ensure that all articles met two inclusion criteria that were added slightly later in the process:

1) Legitim* in the abstract must not be used interchangeably with another term.
2) Articles that deal only with health or technology but not both are subjected to the higher level of inclusion criteria for legitimacy theorization.

Analysis

Example

Within STS, codes that dealt with discourse and narrative (such as “scientific discourse” and “assumption: legitimacy is discourse-based”) were consolidated into a thematic code “discourse.” Consolidating all “discourse” codes made it easier to capture co-occurrences of thematic codes such as “negotiation” or “boundary work,” without losing the integrity of the initial coding process in Atlas.ti when transitioning coding to Excel. The Excel transfer made it easier to see connections among the codes more clearly, as

noise was reduced. When a code co-occurrence was unexpected or did not fit, AA returned to the original text to further flesh out and edit the thematic codes as needed. This information was included in the disciplinary summaries, which later became the results section of this article.
