# Supplementary material for: Legitimacy as Social Infrastructure: A Critical Interpretive Synthesis of the Literature on Legitimacy in Health and Technology
Source: JMIR Hum Factors. 2025 Mar 5;12:e48955. doi: 10.2196/48955 (PMC11923462; doi:10.2196/48955)

Multimedia Appendix 2
Howe et al. (2025) Legitimacy as Social Infrastructure: A Critical Interpretive Synthesis of Literature on Legitimacy in Health and Technology

Multimedia Appendix 2
Example, Disciplinary Network Visualization

Discipline: STS
Produced within Atlas.ti

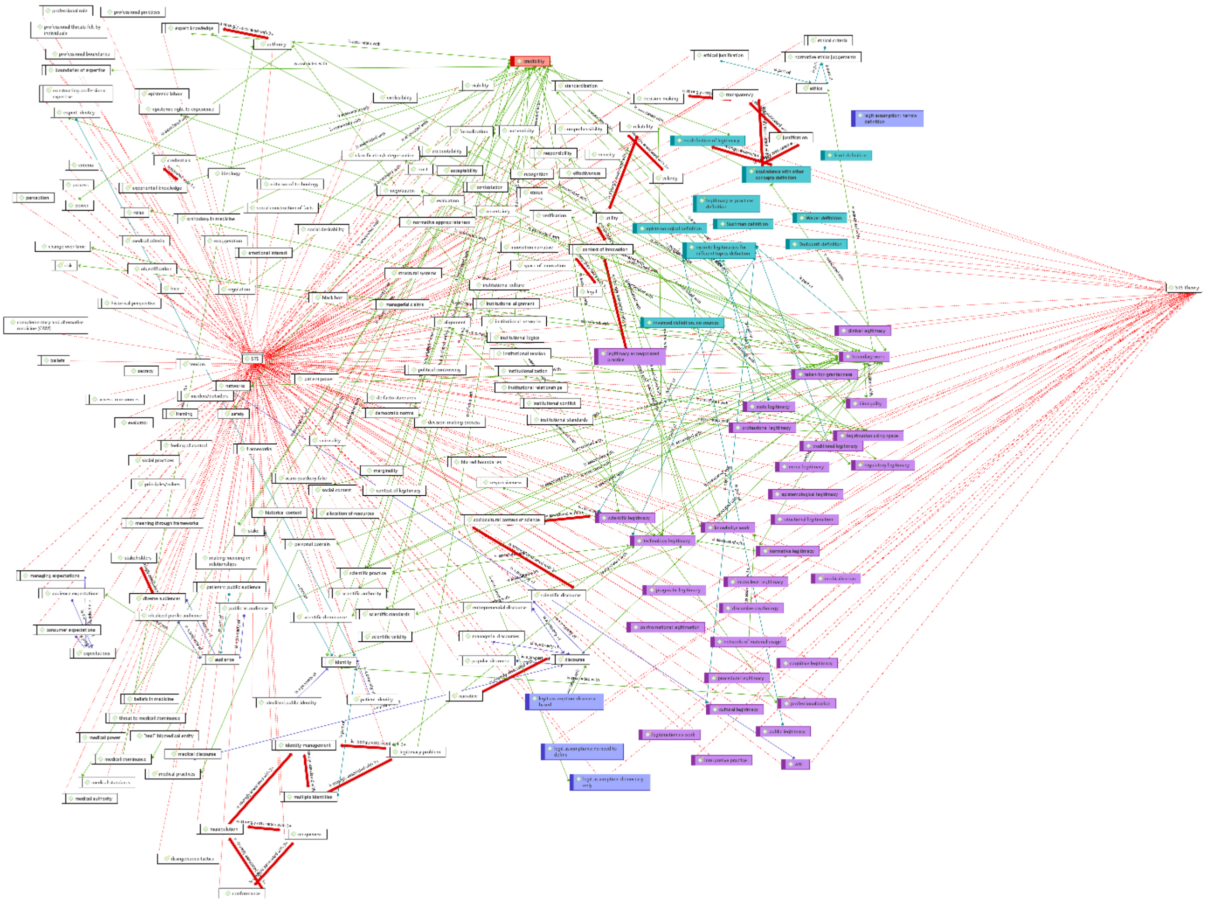

Supplement: Multimedia Appendix 2 [file humanfactors_v12i1e48955_app2.pdf]
